# Electrochemistry and capillary condensation theory reveal the mechanism of corrosion in dense porous media

**DOI:** 10.1038/s41598-018-25794-x

**Published:** 2018-05-09

**Authors:** Matteo Stefanoni, Ueli M. Angst, Bernhard Elsener

**Affiliations:** 1 Institute for Building Materials, ETH Zurich, Stefano-Franscini-Platz 3, Zurich, CH-8093 Switzerland; 2 University of Cagliari, Department of Chemical and Geological Science, I-09100 Monserrato, CA Italy

## Abstract

Corrosion in carbonated concrete is an example of corrosion in dense porous media of tremendous socio-economic and scientific relevance. The widespread research endeavors to develop novel, environmentally friendly cements raise questions regarding their ability to protect the embedded steel from corrosion. Here, we propose a fundamentally new approach to explain the scientific mechanism of corrosion kinetics in dense porous media. The main strength of our model lies in its simplicity and in combining the capillary condensation theory with electrochemistry. This reveals that capillary condensation in the pore structure defines the electrochemically active steel surface, whose variability upon changes in exposure relative humidity is accountable for the wide variability in measured corrosion rates. We performed experiments that quantify this effect and find good agreement with the theory. Our findings are essential to devise predictive models for the corrosion performance, needed to guarantee the safety and sustainability of traditional and future cements.

## Introduction

Corrosion of metals in porous media is a phenomenon that occurs in a variety of conditions, including metals in contact with soils^1^, in transportation and storage of granular materials^2,3^, or in timber^4,5^. Another example of particular scientific and practical relevance is corrosion of reinforcing steel in concrete. This is because reinforced concrete is worldwide among the most widely used materials^6^. Although generally durable in many different exposure conditions, corrosion of the reinforcing steel embedded in the cement-based material is the most frequent cause of premature degradation^7,8^ and causes high costs, comparable to those related to weather and climate disasters^9^. The pore system of concrete presents a unique environment to the steel, both in terms of pore size distribution – pore sizes ranging over several orders of magnitude and reaching down to the nanometer scale^10^ –, and in terms of electrolyte chemistry, that is, electrolyte of high ionic strength and highly alkaline pH^11^.

A relevant cause for corrosion of steel in concrete is carbonation of the concrete^7^, that is, the loss of alkalinity. In sound concrete, pH values are initially in the range of 12.5–14^6,7^. Upon chemical reactions of the cement paste with carbon dioxide, penetrating the pore system of the concrete from the exposure environment, the pH may decrease to levels around 8–9^12^. It is well known that this leads to a relatively uniform type of corrosion attack on the reinforcing steel^7^.

The mechanism governing the rate of this corrosion process has been subject to investigations since the 1950s^13,14^. A number of different hypotheses have been suggested including kinetic control related to the electrical resistivity (inverse of conductivity) of the concrete or the limited availability of oxygen at the steel surface – that both correlate with the moisture state of the concrete pore system. There is however, no consensus on the mechanistic understanding. This is one of the main reasons for the general lack of reliable, quantitative models for the prediction of corrosion rates in carbonated concrete^14^, which presents a severe obstacle for holistic life cycle assessments of both traditional and novel building materials.

The question of corrosion rates in carbonated concrete is, after having been addressed for over half a century, currently receiving more and more attention. This is because of the increasing market share and promotion of blended cements as alternatives for the traditional Portland cement^15–17^. These modern cement types are claimed to be more environmentally friendly, thanks to the reduced energy consumption as well as reduced emissions of greenhouse gases such as carbon dioxide during production. Their ability, however, to protect the embedded reinforcing steel from corrosion is still under debate. For holistic assessments of the sustainability of these modern materials, the long-term corrosion performance is crucial. Considering the time to corrosion initiation alone will not permit realistic assessments, because some of the modern binders are known to carbonate substantially faster than Portland cement^18–20^; thus, it is important to also consider the corrosion propagation stage, which requires the knowledge and prediction of corrosion rates. Finally, the continuously increasing diversity in physical and chemical properties of modern cement types imposes an urgent need for a mechanistic model for the prediction of corrosion rates in carbonated concrete^21^.

**Figure 1:**
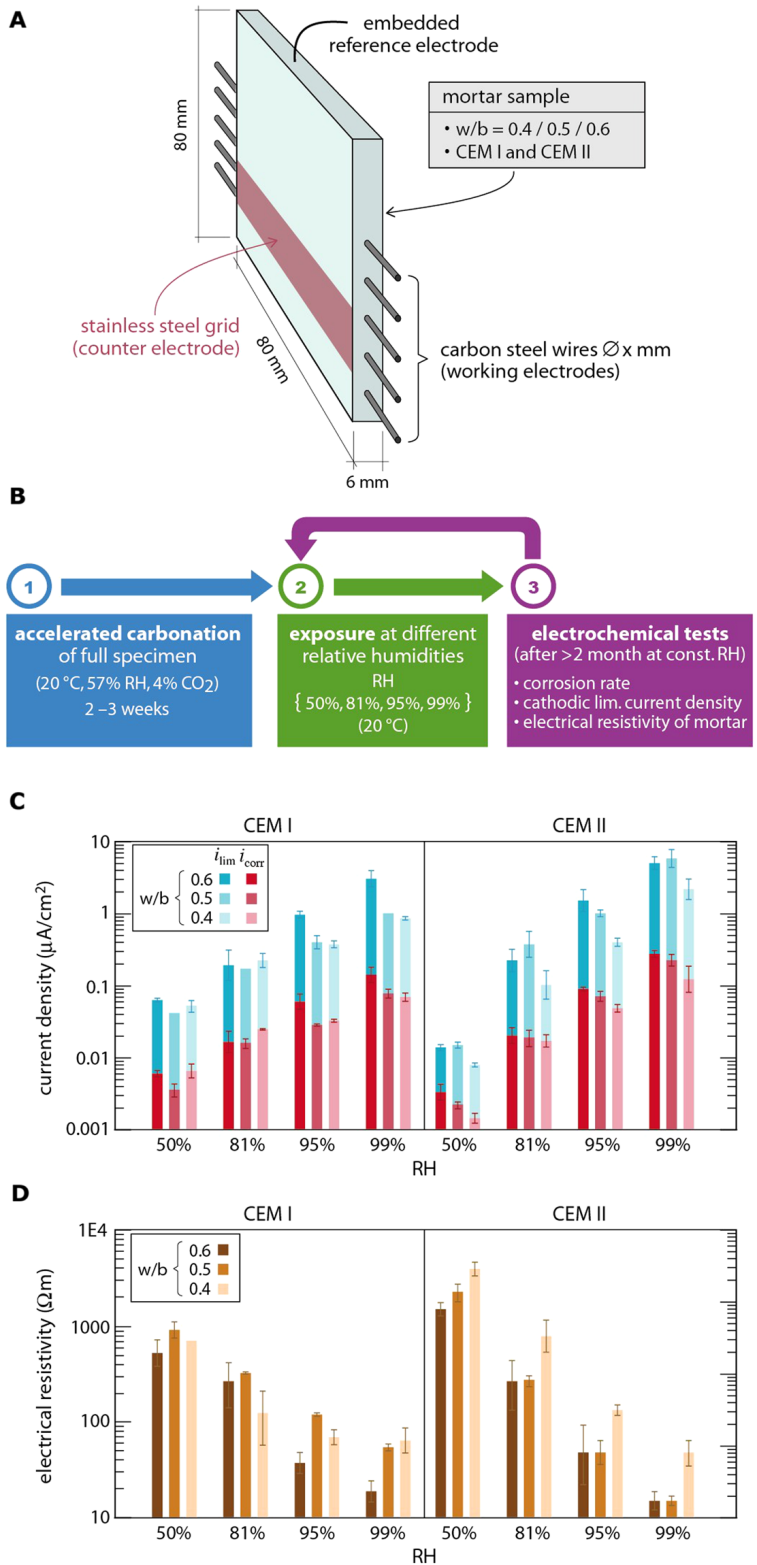
Effect of relative humidity (RH) on corrosion rate and other electrochemical parameters in carbonated mortar. (**A**) Sample geometry and instrumentation with embedded steel wires and sensors (Supplementary Figures 1 and 2); **(B)** Experimental procedure including initial carbonation followed by exposure at different RH and corresponding electrochemical measurements; **(C)** Cathodic limiting current density (*i*_lim_) and corrosion rate (*i*_corr_) for the different studied systems (water/binder ratios and cement types) as a function of RH (Supplementary Table 1); **(D)** Electrical mortar resistivity as a function of material parameters and RH (Supplementary Table 1).

Here, we propose a new mechanism to explain the corrosion rate in carbonated cement-based materials, as a model for dense porous media with a near neutral pore solution (pH ≈ 8–9). Our mechanism combines the capillary condensation theory with corrosion science and electrochemistry. We designed a novel experimental setup^22,23^ that permits studying the relevant electrical and electrochemical parameters and that allows for fast equilibration with exposure conditions, that is, carbon dioxide and relative humidity (RH) (Fig. 1A) (Supplementary Figures 1 and 2). The main advantage of this is that it permits studying the corrosion process under well-defined conditions, which is not possible in traditional setups that typically include gradients in moisture, carbonation state, and other relevant properties. Various samples were prepared, including two different cement types and three different water/binder (w/b) ratios. After pre carbonation, the corrosion process was studied at different RH by means of a number of electrochemical measurements (Fig. 1B).

## Results

### Corrosion rate depends mainly on relative humidity

The corrosion rate of steel in carbonated mortar increased dramatically with increasing RH (Fig. 1C and Supplementary Table 1). Changing RH from 50% to 99% raised the corrosion rate by up to two orders of magnitude (Supplementary Figure 3). Compared to this, the water/binder ratio and the cement type were found to be minor influencing factors. These affected the corrosion rate generally by a factor of up to 2–3 (Supplementary Figure 3). Similarly, the cathodic limiting current density (*i*_lim_) increased strongly with RH, and was moderately affected by water/binder ratio and cement type (Fig. 1C, Supplementary Table 1 and Supplementary Figure 3). Finally, the impact of these influencing factors was also comparable for the electrical resistivity of the mortar (Fig. 1D, Supplementary Table 1 and Supplementary Figure 3).

**Figure 2:**
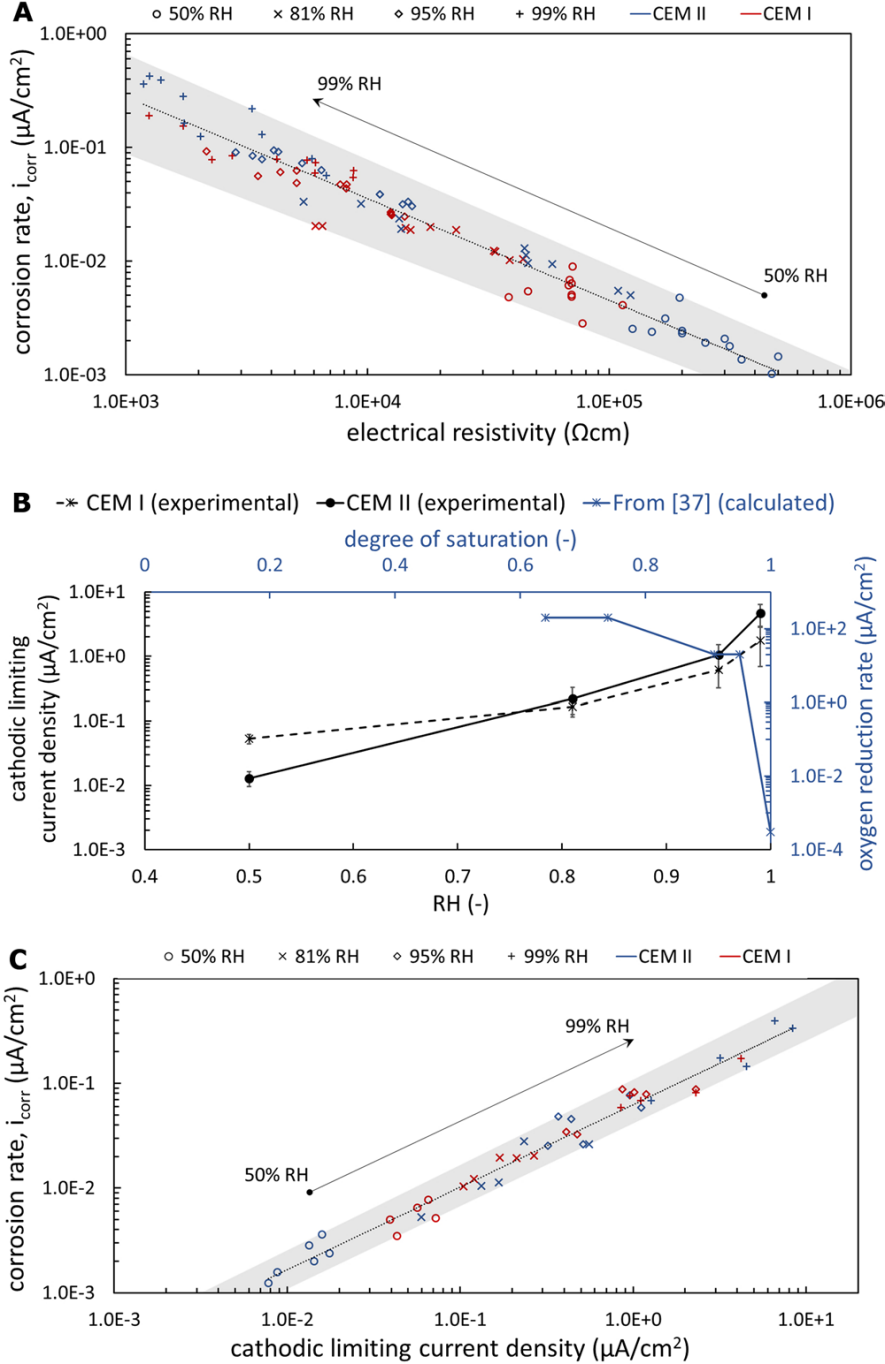
Correlation between corrosion rate (*i*_corr_) and other electrochemical parameters as well as illustration of the oxygen paradox in dense porous media. (**A**) Traditional representation of *i*_corr_ vs. electrical resistivity of concrete that may erroneously be interpreted as proof for a causality; (**B**) The cathodic limiting current density has been experimentally measured as increasing with RH (black series: symbols = mean values, whiskers = standard deviations). On the contrary, in the literature, oxygen has always been considered as decreasing with RH, because of argumentations only based on the purely physical diffusion behaviour. An example taken from the literature^37^ is reported in the figure, in order to show the completely opposite trend between what has been measured (black series) and what has been calculated in^37^ (blue series); (**C**) Close correlation between *i*_corr_ and *i*_lim_. Such correlation has never been shown before and it suggests there is the same mechanism controlling the two electrochemical processes.

There exists a relationship between the corrosion rate and the electrical resistivity of the mortar as shown in Fig. 2A. This is a common form of representing the data^24–30^, which may ultimately be traced to the widespread hypothesis of a causality between these two parameters. Several authors^24–29^ suggested that the ohmic resistance of the mortar is the rate-limiting step in the corrosion process, that is, the ion transport between anodic and cathodic sites (through the pore system of the mortar) limiting the corrosion current. However, this mechanistic explanation has been criticized on an experimental basis, pointing out that ohmic control is in contrast with the correlation found between corrosion rate and corrosion potential^30,31^. Additionally, ohmic control is also unlikely from a theoretical viewpoint, because the uniform type of corrosion, typically observed in carbonated concrete, means that both the anodic and cathodic reactions occur homogeneously and time-variably distributed over the steel surface, forming micro-cells with negligibly small ohmic resistance due to the microscopic distance between the neighboring reaction sites^32–34^.

### The role of the cathodic reaction and the oxygen paradox

Figure 1C and Supplementary Figure 4 show that the cathodic limiting current density was always one order of magnitude higher than the corrosion rate, regardless of the experimental parameters studied. The measured *i*_lim_ indicate the maximum achievable cathodic reaction current density, which is dictated by the availability of oxygen at the steel surface. These results illustrate that the availability of oxygen at the steel surface was here never a limiting factor for the corrosion rate.

Figure 2B shows the dependency of *i*_lim_ on RH. Interestingly, *i*_lim_ increases significantly with increasing RH, that is, the limiting current density increases over approximately 2 orders of magnitude when changing RH from 50% to 99%. This contradicts literature stating that oxygen diffusion is hindered by higher pore water saturation states (higher RH) as a consequence of the difficulties of oxygen to diffuse through water filled pores with respect to empty pores^35–40^. Figure 2B shows an example of estimated maximum oxygen reduction rates based on oxygen diffusion calculations as a function of RH^37^. It can be noted that this trend is opposite the present experimental observation. We suggest that this apparent contradiction can be explained largely by the area fraction of the steel surface that is in contact with electrolyte. This will be addressed in more detail in the discussion.

Nevertheless support for this hypothesis can also be found in the observation that *i*_corr_ and *i*_lim_ reacted consistently on changes in environmental conditions and depended also in a similar manner on sample-specific conditions (Supplementary Figure 4). This is also apparent from the correlation of the two parameters shown in Fig. 2C.

## Discussion

### Mixed anodic and cathodic control

The corrosion process is controlled by the kinetics of both the anodic and cathodic partial reactions. This may be represented with the well-known Evans diagram^7,41^, an example of which is shown in Supplementary Figure 5. As explained earlier, a resistive (ohmic) limitation of *i*_corr_ can be neglected (due to the uniformity of the corrosion process) and the cathodic reaction is, in the potential range relevant here, not limited by the transport of oxygen (Fig. 1C). Thus, the anodic and cathodic reaction kinetics are essentially defined by their reversible potentials (*E*_a,rev_ and *E*_c,rev_), their exchange current densities (*i*_a,o_ and *i*_c,o_), and their Tafel slopes (*β*_a_ and *β*_c_)^42^. These parameters depend essentially on the chemistry of the electrolyte and on the metal. Variations in these parameters may, in principle, affect the corrosion rate. However, as will be shown below, it is highly unlikely that in the present case, the marked variations in corrosion rate displayed in Fig. 1C were caused by changes in these electrochemical parameters.

In this study, the main influencing variable for *i*_corr_ was the relative humidity (Fig. 1, Supplementary Figure 3). The relative humidity mainly affects the water content in the mortar pore system. While this influences the amount of water present at the steel surface, it has a secondary influence on the chemical composition of the pore solution. This is because of the extremely high solid/liquid ratio, which keeps the concentrations of most species in the electrolyte close to or at saturation. Thus, concentrations of oxygen and ferrous ions as well as pH are expected to be negligibly affected by RH. Nevertheless, even if minor concentration changes were possible, they can impossibly explain the factor of 200 measured between corrosion rates at 99% and 50% RH. This is because it would mean a concentration difference of ferrous ions of 18 orders of magnitude, a pH difference of 9 units, or an oxygen concentration difference of 35 orders of magnitude (Supplementary Note 1).

Thus, for a given sample (given binder type, w/b ratio, and type of steel), the reversible potentials, the exchange current densities, and the Tafel slopes of the anodic and cathodic partial reactions are expected to be similar for all RH. Consequently, the corrosion current density should also be similar for all RH, here denoted ${i}^{\sim }$ (Supplementary Figure 5). Ge *et al*. determined the effect that variation of exchange current densities, reversible potentials and Tafel slopes would have on the corrosion rate based on the data found in literature^43^. They showed that these parameters, as per the scatter found in the literature, can lead to a corrosion rate variation of a maximum factor of 3.

In summary, the high variations of observed corrosion rate when changing RH (Fig. 1C) can neither be explained by the electrochemistry of the corroding system, nor by kinetic limitations arising from the ohmic resistivity of the pore system, nor by limited oxygen availability. Our hypothesis is that a simple area effect, arising from RH-depending steel surface wetting in the concrete pore system, can explain these variations in observed corrosion rate.

### The electrochemically active area controls the corrosion current

The moisture state of concrete has an influence on the portion of the steel area that is in contact with liquid water. At low RH, large pores and voids may be air-filled with only a few monolayers of adsorbed water present on the steel surface^44^. In these thin films of water, electrochemical reactions are strongly limited, among other reasons because any transport of charged species involved in the anodic and cathodic processes is hardly possible^45–47^. Capillary pores, on the other hand, may according to the capillary condensation theory^48^ be stably filled with liquid water. In pores filled with solution, electrochemical reactions will be enabled to occur in a similar manner as in a bulk solution.

The condensation of water in porous systems can be described by the Kelvin-Laplace equation^49–51^. This equation describes both the stable meniscus, *r*, and the pressure in the liquid phase, *p*^l^, as a function of RH:

1 $$r=-\,\frac{2{\sigma }^{lg}{v}^{l}}{\mathrm{ln}(RH)\cdot RT}\propto -\,\frac{1}{\mathrm{ln}(RH)}$$

2 $${p}^{l}=-\,\frac{RT}{{v}^{l}}\,\mathrm{ln}(RH)\propto -\,\mathrm{ln}(RH)$$

**Figure 3:**
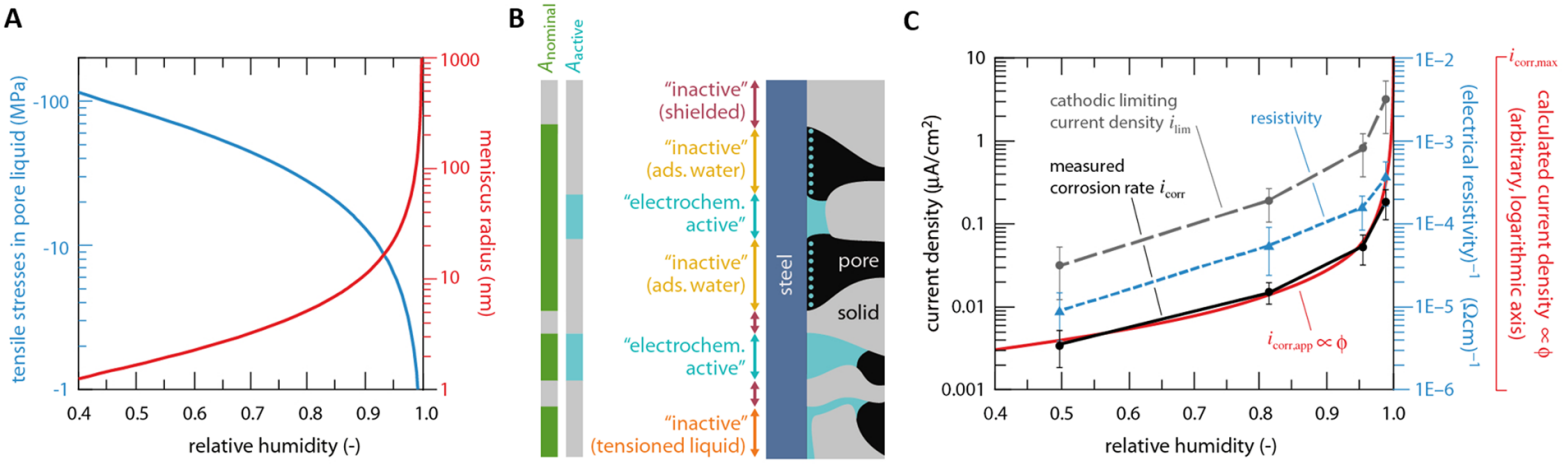
The amount of electrolyte available for electrochemical reactions at the steel surface depends strongly on RH and determines the apparent corrosion current density. (**A**) Tension of concrete pore liquid and radius of stably water filled pores as a function of RH, illustrating that with increasing RH, the amount of pore liquid behaving as electrolyte increases significantly; (**B**) schematic illustration of the electrochemically active steel surface in dense porous media depending on the porosity and the presence of electrolyte; green is the nominal area, that is the steel surface area not shielded by adherent solid phases; (**C**) Measured corrosion rate, cathodic limiting current density and the inverse of the electrical resistivity of the mortar as a function of RH (markers = average values, whiskers = standard deviations; based on all measured data). The predicted apparent corrosion current density (red), based on the capillary condensation theory and the concept of the electrochemically active area fraction, is in excellent qualitative agreement with these three parameters. Note the logarithmic scale, meaning that several orders of magnitude are expressed in this graph, following a distribution closely in agreement with how the electrochemically active area ratio (*ϕ*) is supposed to vary as a function of RH (red curve = qualitative plot of eq. (3)).

**Figure 4:**
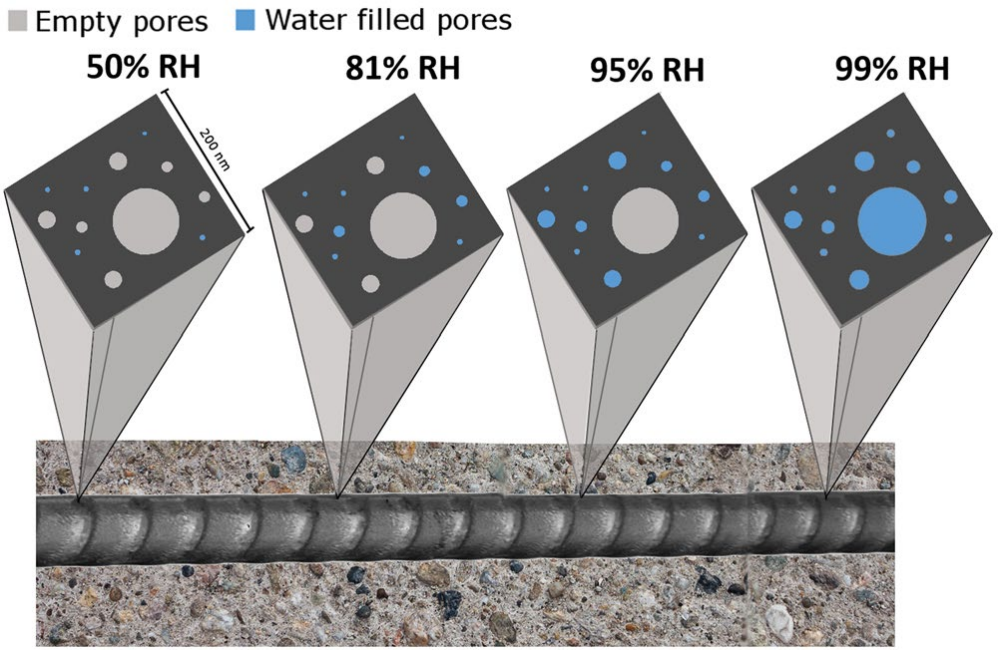
Graphical representation of the changing steel surface area in contact with water. As a consequence of water condensation in pores at different relative humidity, the steel area on which corrosion can take place varies nonlinearly with the RH.

Here, *r* can be considered the maximum stably water-filled pore radius, ${\sigma }^{lg}$ is the surface tension of water-air, ${v}^{l}$ is the molar volume of water, *R* is the ideal gas constant, and *T* is the temperature. Figure 3A illustrates the strongly nonlinear nature of these relationships. Cementitious materials are characterized by a wide pore size distribution (from approx. 1 nm up to the micrometer scale), which means that as the relative humidity varies, a different volumetric fraction of this porosity is water-filled. At low to intermediate RH, the pore liquid may in small pores at the steel-concrete interface be under considerable tension and not be able to behave as a liquid^52–54^. Only at high RH (>80%) is the tension reduced to a level approaching bulk solution behavior. At the same time, the radius of stably water-filled pores increases strongly nonlinearly with increasing RH. As a consequence of these two facts, the area of steel surface being in contact with liquid water capable of behaving as an electrolyte, *A*_active_, can be expected to increase also nonlinearly with RH. The result is a nonlinear increase of the water filled porosity, which determines an increase of the steel surface area being in contact with liquid water (Fig. 4).We suggest the following proportional relationship between area fraction $\varphi$ and RH:

3 $$\varphi =\frac{{A}_{active}}{{A}_{nominal}}\propto -\,\frac{1}{\mathrm{ln}(RH)}$$

Here, *A*_nominal_ is the surface area of the steel in contact with pores and voids in the concrete (not shielded by adherent solid phases), and *A*_active_ is the fraction of this area at which electrochemical processes can occur (Fig. 3B). We acknowledge that the transition from electrochemically “inactive” areas to “active” areas is gradual (Fig. 3A). Nevertheless, the concept of introducing an area fraction according to eq. (3) is considered a valid approach to take into account the fact that a significant portion of the steel surface is, below saturation, not in conditions that permit the occurrence of electrochemical reactions (because it is either only wetted with a few monolayers of adsorbed water or because the water held in gel pores is under considerable tension).

Figure 3C provides evidence for this suggested approach (eq. 3), as the concept of an electrochemically active area fraction $\,\varphi$ can very well explain the large experimentally observed variations (Fig. 1C) in measured corrosion current density *i*_corr_. The red curve in Fig. 3C can be expressed as follows:

4 $${i}_{corr,app}={i}_{corr,{\rm{\max }}}\cdot \varphi $$

Here, *i*_corr,app_ means the apparent corrosion rate, that is, the corrosion rate that is experimentally obtained when a measured corrosion current is divided by the total embedded steel surface area *A*_steel_. We suggest that *i*_corr,app_ can be calculated as the product of the maximum corrosion current density, hypothetically achievable at full saturation of all pores, and the area ratio $\varphi$. The maximum achievable corrosion current density is.

5 $${i}_{corr,{\rm{\max }}}=\frac{{i}^{\sim }\cdot {A}_{{nominal}}}{{A}_{steel}}$$

Here, ${i}^{\sim }$ indicates the corrosion current density determined by the anodic and cathodic reaction kinetics. As discussed above, these are largely independent of RH, thus ${i}^{\sim }$ is here assumed a constant. An exception to this may be the influence of the ferrous ion concentration at the steel surface affecting the electrode kinetics, or prolonged (as opposed to short-term) saturation, leading to limited oxygen availability. This will be addressed in more detail below.

Figure 3B also shows a qualitative correspondence with *i*_lim_ as well as with the inverse of the electrical resistivity. This can be explained by the fact that these two parameters are directly related to the electrochemically active steel surface area.

### Implication for corrosion of metals in porous media

We believe that the here exposed theory presents a major step ahead in the understanding of electrical and electrochemical processes in unsaturated porous media, particularly of dense porosity, that is, pore sizes where capillary condensation is possible. While it is well known that water – in conditions able to behave as an electrolyte, thus permitting transport of ions – is essential for electrical and electrochemical processes to occur, the related effects of the degree of pore saturation and tension in the capillary water, are typically not considered in studies addressing corrosion rates in porous media.

Models for the prediction of corrosion rates of steel in carbonated concrete are mainly based on the assumption that oxygen reduction is the rate-limiting step of the process^37,40,55^, or on a calculation of corrosion rate from oxygen concentration and resistivity^56^. As shown in this article, the parameters that are at the base of these models can, however, not explain the behavior and magnitude of the corrosion process in unsaturated porous media, where the cathodic limiting current density appears to be always 10 times higher than the corrosion rate – even at high RH such as 99%. We do not generally exclude that oxygen diffusion through the concrete cover may become a rate limiting step. However, for this to occur, extremely long and permanently fully saturated conditions are needed until all dissolved and gaseous oxygen (present in air voids within the concrete), becomes consumed and further supply is possible only from the exterior of the concrete. In typical carbonation exposure conditions, the moisture state of the pore system of concrete is below saturation. This is because carbonation is fastest around average RHs of 60–70%^7^. Under conditions of prolonged wetting (e.g. submerged conditions), carbonation typically occurs at negligibly low rates^57^ and does hardly reach the steel depth within the service life of a structure. Thus, in exposure conditions that favor carbonation, such as constantly relatively dry conditions (60–70% RH) or cyclic wetting/drying exposure, a substantial portion of the pores in the concrete is always filled with air, and thus oxygen. As a consequence, we consider the limitation of oxygen availability at the steel surface by transport, that is diffusion exclusively through the liquid phase of the pore system (as predicted in Fig. 2, blue line), hardly relevant for corrosion in carbonated concrete in the vast majority of practical situations.

In addition to models for the corrosion rate, the here suggested approach might greatly influence the conception of corrosion-induced concrete cracking. Acknowledging that it is the steel area fraction on which corrosion processes occur, which changes dramatically upon RH changes, rather than the actual corrosion current density (the corrosion rate per unit wet and electrochemically active area, ${i}^{\sim }$), has dramatic consequences for the distribution of corrosion products and the consequent localization of stresses due to the crystallization in the porous system. Concrete cracking due to precipitation of corrosion products cannot be simulated by models based on uniform corrosion assuming release of corrosion products into the concrete pore system homogeneously distributed over the entire steel surface.

The main strength of our theory can be found in its simplicity. Predictions of corrosion kinetics in dense porous media rely on just two conceptual aspects: i) the inverse proportionality between the electrochemically active steel surface ratio ($\varphi$) and the logarithm of the relative humidity (eq. 3), grounded on the theory of capillary condensation, and ii) the corrosion current density ${i}^{\sim }$ that, according to corrosion science, establishes depending on metal surface properties and electrolyte chemistry – however largely independent of RH. Further work should aim at quantifying the proportionality constant needed for eq. (3), on the basis of material properties, such as porosity and pore size distribution, while electrochemistry and sound corrosion science can deliver ${i}^{\sim }$. To take into account that the microstructure of the concrete at the steel-concrete interface may differ from regions further away^58^, further work should also characterize the porosity and pore size distribution of the volume that directly controls the water distribution and the electrolyte chemistry at the steel surface.

### Concluding remarks

The experimental results presented in this work show that the by far most dominant factor influencing the corrosion rate in carbonated concrete is the moisture state. A fundamentally new approach – combining the capillary condensation theory with electrochemistry – was proposed to explain the scientific mechanism of corrosion kinetics in dense porous media. It was shown that capillary condensation in the pore structure defines the electrochemically active steel surface, the variation of which explains the orders of magnitude over which the measured corrosion rates span.

The proposed conceptual model lays the basis for reliable predictions of corrosion rates in various dense porous media in a multitude of exposure conditions. For the case of reinforcing steel corrosion in carbonated concrete, to guarantee safety, serviceability, and sustainability of engineering structures, enhancing the predictions of the performance in actual exposure conditions is more urgently needed than ever, mainly due to the continuing loss of alkalinity in modern and future cement types that was discussed in the introduction.

## Materials and Methods

### Test sample specifications

The samples (dimension 80 × 80 × 6 mm^3^, Fig. 1A) were cast with embedded carbon steel (St 37) wires (diameter = 0.5 mm) serving as working electrodes, a stainless steel grid (10 × 100 × 1 mm^3^), to be used as counter electrode, and an embedded Ag/AgCl sensor acting as reference electrode for the electrochemical tests^22,23^ (Supplementary Figures 1 and 2). In total, 4 samples per kind were prepared, each of them allowed for 2 different measurements of corrosion rate and resistivity and 1 measurement of cathodic limiting current. That means the averages and standard deviations are made out of 8 measurements for corrosion rate and matrix resistivity and 4 measurements for the cathodic limiting current.

### Mix design

In order to evaluate the influence of different pore structures, 6 different types of specimens were tested. These were produced by varying the binder type and the water to binder ratio (w/b); two different cements and three different w/b ratios were used: the binders CEM I 52,5 R and CEM II/B-M (T-LL) 42,5; the w/b ratios were 0.4, 0.5 and 0.6.

The mix design was chosen to allow the best fluidity while maintaining a high stability of the cementitious suspension. The sand/binder ratio was 2 and the sand had a maximum particle diameter of 1 mm. A poly-carboxylate ether superplasticizer with de-foaming agent was added to the mixes with w/c 0.4 and 0.5 (0.6% and 0.2% by weight of binder, respectively) in order to increase the fluidity and thus permitting filling the mould; the amount was chosen in order to achieve a visually similar fluidity of the mortars. The specimens were demoulded after one day and cured at 95% RH for 7 days before being carbonated.

### Carbonation procedure

The samples were carbonated in a carbonation chamber at 20 °C, 57% relative humidity and 4% CO_2_ concentration. The time required for complete carbonation was:

- 3 to 7 days for CEM II mortars;
- 7 to 14 days for CEM I mortars.

These durations were obtained by the phenolphthalein test on companion samples, prior to this experimental study. The phenolphthalein indicator allows for a rough evaluation of the pH value and it is widely used in carbonation studies in the field of cementitious materials;^59^ in a non-carbonated sample the indicator gives a bright purple color, which indicates the pH is higher than 9.9. In the interval 8.0–9.9 the coloring shades away, until it disappears below pH 8. In this study the carbonation process is not under evaluation, but complete carbonation of the material is needed in order to trigger the corrosion process. To ensure complete carbonation of the samples used for the present study, a safety extra-time in the carbonation chamber was applied: CEM II mortars were carbonated for 2 weeks and CEM I mortars for 3 weeks.

### Exposure conditions

After carbonation, the samples were studied in different exposure conditions of controlled and constant environments (Fig. 1B): 50% RH, 81% RH, 95% RH and 99% RH; at a constant temperature of 20 °C. The relative humidity was controlled by means of either climatic conditioning rooms (50% RH and 95% RH) or of saturated Ammonium Sulfate solution (81% RH) or ultrapure water (99% RH), taking care no water condensation would take place in the container by keeping the temperature constant.

### Time to equilibration

Monitoring of the mass of the samples showed a weight equilibration for all the different samples, at a constant relative humidity, after 1 to 2 weeks. The measurements of all the parameters started after minimum 2 months of exposure at a constant relative humidity, once stable conditions were achieved (Fig. 1B).

### Electrochemical tests

All the electrochemical tests were performed using a potentiostat Metrohm Autolab PGSTAT30. The embedded Ag/AgCl sensor was always used as reference electrode and its reference potential was checked by means of an external Ag/AgCl reference electrode. One carbon steel wire was used as working electrode and the stainless steel grid was used as counter electrode. The measurements were repeated over time for each exposure condition. The electrolyte of the electrochemical system is the aqueous phase condensed in the pore structure in equilibrium with environmental humidity.

Corrosion rate: the instantaneous corrosion current density was determined by polarization resistance measurements. The polarization resistance R_p_′ of the single steel wires was measured at ± 10 mV around the open circuit potential with a scan rate of 0.1 mV/s. The *IR*-drop in the mortar was taken into account indirectly. Impedance measurements (see below) were performed right before each polarization resistance test, and the obtained ohmic resistance R_Ω_ was subtracted from the total resistance R_p_′ to get the *IR* drop-free polarization resistance values R_p_.

The corrosion rate, *i*_corr_, was then calculated using the following equation:

6 $${i}_{corr}=B/{R}_{p}$$

Where B is a parameter depending on the electrochemical properties of the considered system; for iron in actively corroding state a value of 26 mV is commonly used.

Ohmic resistance R_Ω_: in the same electrode configuration as for the polarization resistance tests, the electrical resistance was measured by means of electrochemical impedance spectroscopy (50 frequencies logarithmically distributed between 10^5^ and 1 Hz, voltage amplitude = 10 mV). The value of ohmic resistance R_Ω_ was extrapolated by fitting of the first semicircle appearing in the Nyquist plot and considering the value first intercepted on the real impedance axis.

Electrical resistivity: the resistivity of the mortars was calculated by converting R_Ω_ to resistivity with a cell constant (0.05 cm) that was determined experimentally, that is, by measuring the ohmic resistance between two disk electrodes placed on the facing sides of the mortar samples.

Cathodic limiting current density: cathodic potentiostatic polarization tests were carried out by imposing a potential 500 mV cathodic to the open circuit potential. The resulting current decay was monitored for 30 minutes, at the end of which the steady state current was recorded. For this test, a different steel wire of the sample was used, that was not used for corrosion rate measurements in order not to compromise the surface state. In this case the *IR*-drop compensation was directly applied as an input to the polarization procedure, the value was set as 80% of the extrapolated ohmic resistance from impedance measurement carried out using the electrode configuration as for the cathodic polarization.

### Data availability

The experimental data supporting the findings of this study and used to create the figures are available in the paper’s Supplementary Information. All the other data are available from the corresponding author(s) upon request.

## Electronic supplementary material

Supplementary Information
