# Supplementary material for: Electrochemistry and capillary condensation theory reveal the mechanism of corrosion in dense porous media
Source: Sci Rep. 2018 May 9;8:7407. doi: 10.1038/s41598-018-25794-x (PMC5943290; doi:10.1038/s41598-018-25794-x)
Supplement: Supplementary file 1 — Supplementary Information [file 41598_2018_25794_MOESM1_ESM.pdf]

Electrochemistry and capillary condensation theory reveal the mechanism of corrosion in dense porous media

Matteo Stefanoni[1], Ueli M. Angst[1,*], Bernhard Elsener[1,2]

[1] Institute for Building Materials, ETH Zurich, Stefano-Franscini-Platz 3, Zurich CH-8093, Switzerland.

[2] University of Cagliari, Department of Chemical and Geological Science, I-09100 Monserrato (CA), Italy.

* Correspondence should be addressed to U. M. Angst. (email: uangst@ethz.ch)

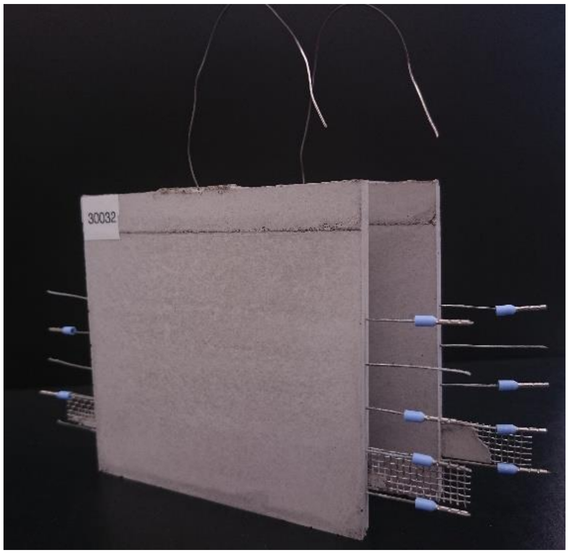

Supplementary Figure 1. Two samples, one behind the other. It is possible to clearly see the 5 embedded carbon steel wires, the stainless steel grid towards the bottom of the sample and the Ag/AgCl reference electrode in the top part. All the necessary electrodes are already embedded in an 80x80x6 mm^2 mortar specimen, resulting in an approximate weight of 80g.

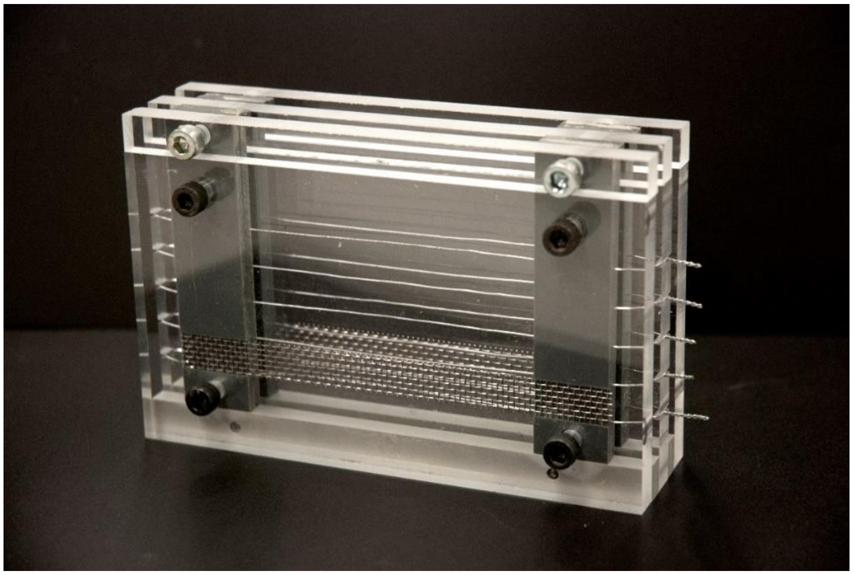

Supplementary Figure 2. Mould specifically designed for the production of the samples. The wires and the grid are tightly placed and tensioned ensuring a defined and reproducible position.

Supplementary Table 1. Mesured electrical and electrochemical paparemetrs. Compilation of average measured values (out of minimum 4 measurements for corrosion rate and electrical resistivity, 2 measurements for cathodic limiting current density) and relative standard deviations. The data are reported divided per type of binder, water to binder ratio and exposure condition used. The same data are reported graphically in Figures 1c and 1d of the manuscript.

	Binder	w/b	Exposure condition			
			50% RH	81% RH	95% RH	99% RH
Corrosion rate (μA/cm^2)	CEM I	0.6	5.83E-03 ±7.5E-04	1.48E-02 ±4.5E-03	5.90E-02 ±1.6E-02	1.50E-01 ±4.0E-02
		0.5	3.48E-03 ±8.0E-04	1.22E-02 ±1.4E-03	2.90E-02 ±8.8E-04	8.00E-02 ±9.0E-03
		0.4	6.34E-03 ±1.7E-03	1.99E-02 ±6.4E-04	3.18E-02 ±1.8E-03	8.00E-02 ±9.5E-03
	CEM II	0.6	3.22E-03 ±9.5E-04	1.90E-02 ±9.0E-03	9.30E-02 ±6.1E-03	3.60E-01 ±3.0E-02
		0.5	2.19E-03 ±2.1E-04	1.80E-02 ±7.0E-03	7.20E-02 ±1.3E-02	2.80E-01 ±5.0E-02
		0.4	1.41E-03 ±2.7E-04	8.00E-03 ±3.0E-03	4.90E-02 ±6.2E-03	1.30E-01 ±6.2E-02
Electrical resistivity (Ωcm)	CEM I	0.6	5.53E+04 ±1.3E+04	2.81E+04 ±1.4E+04	3.78E+03 ±1.1E+03	2.00E+03 ±5.7E+02
		0.5	9.55E+04 ±1.8E+04	3.36E+04 ±2.0E+02	1.30E+04 ±7.4E+02	5.84E+03 ±2.1E+02
		0.4	7.01E+04 ±2.2E+02	1.35E+04 ±7.4E+03	7.29E+03 ±1.3E+03	6.94E+03 ±1.9E+03
	CEM II	0.6	1.60E+05 ±2.6E+04	1.15E+04 ±2.1E+03	5.28E+03 ±3.5E+03	1.55E+03 ±3.5E+02
		0.5	2.38E+05 ±4.2E+04	9.63E+03 ±4.2E+03	5.04E+03 ±9.4E+02	1.56E+03 ±1.7E+02
		0.4	4.09E+05 ±7.8E+04	8.36E+04 ±3.3E+04	1.38E+04 ±1.5E+03	4.91E+03 ±1.4E+03
Cathodic limiting current (μA/cm^2)	CEM I	0.6	6.45E-02 ±8.0E-03	3.40E-01 ±1.4E-01	1.04E+00 ±8.0E-02	3.25E+00 ±9.4E-01
		0.5	4.30E-02	1.85E-01	4.17E-01 ±9.6E-02	1.11E+00
		0.4	5.23E-02 ±1.3E-02	3.00E-01 ±6.4E-02	4.03E-01 ±3.5E-02	9.04E-01 ±5.2E-02
	CEM II	0.6	1.46E-02 ±1.3E-03	3.36E-01 ±9.8E-02	1.59E+00 ±7.2E-01	5.57E+00 ±1.0E+00
		0.5	1.59E-02 ±1.6E-03	7.34E-01 ±3.0E-01	1.11E+00 ±8.8E-02	6.17E+00 ±2.2E+00
		0.4	8.28E-03 ±4.8E-04	1.91E-01 ±8.2E-02	4.43E-01 ±3.3E-02	2.23E+00 ±9.6E-01

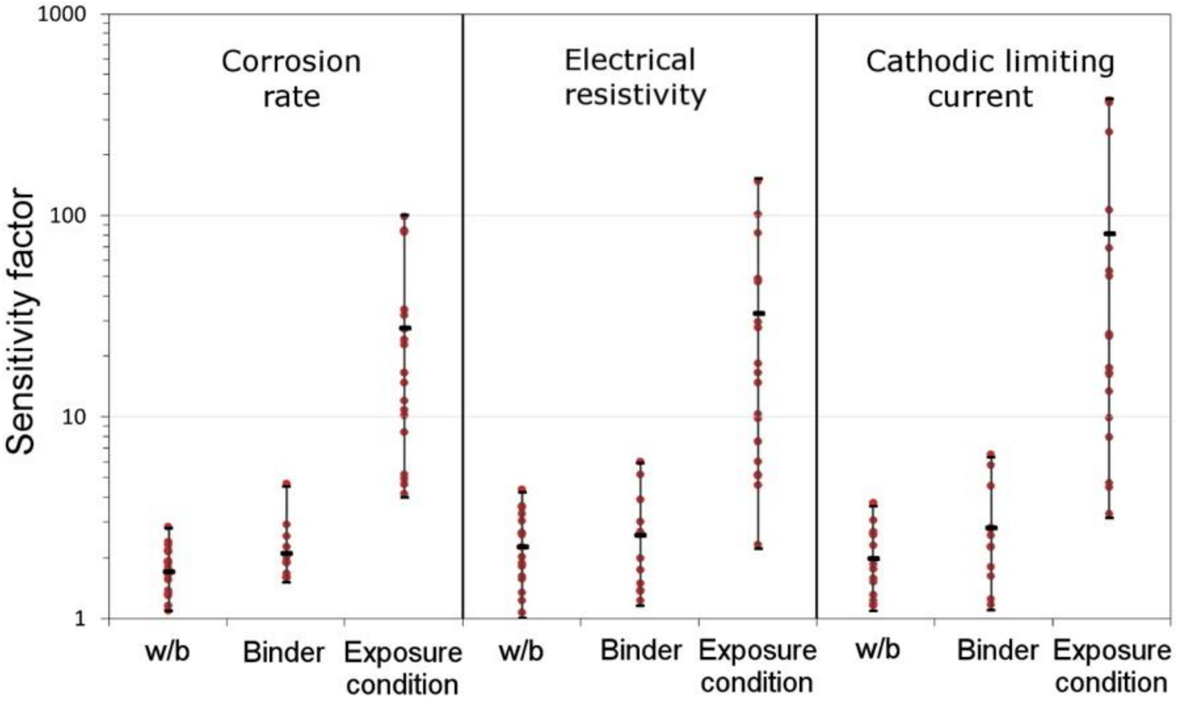

Supplementary Figure 3. Sensitivity factors of the measured parameters to the experimental variables. Ratios between corrosion rate, electrical resistivity and cathodic limiting current average values (reported in Fig. 2 and Supplementary Note 1) have been here used to show the influence on these parameters of the variation of water to binder ratio, binder type and exposure condition. In each case the sensitivity factors of one single parameter are calculated as the ratio between obtained average values (reported in Supplementary Table 1) of the same electrical/electrochemical parameter changing one variable (with the higher value amongst the two always as numerator), keeping the other experimental variables constant. As an example, the sensitivity of corrosion rate to the water to binder ratio is reported as ratios between average corrosion rates of samples of the same binder, stored in the same exposure condition, but realized with different water to binder ratios.

It clearly appears that the exposure RH is by far the most influencing experimental variable for every measured parameter, followed by the type of binder used and the water to binder ratio, these latter two having an overall comparable influence on electrical / electrochemical properties.

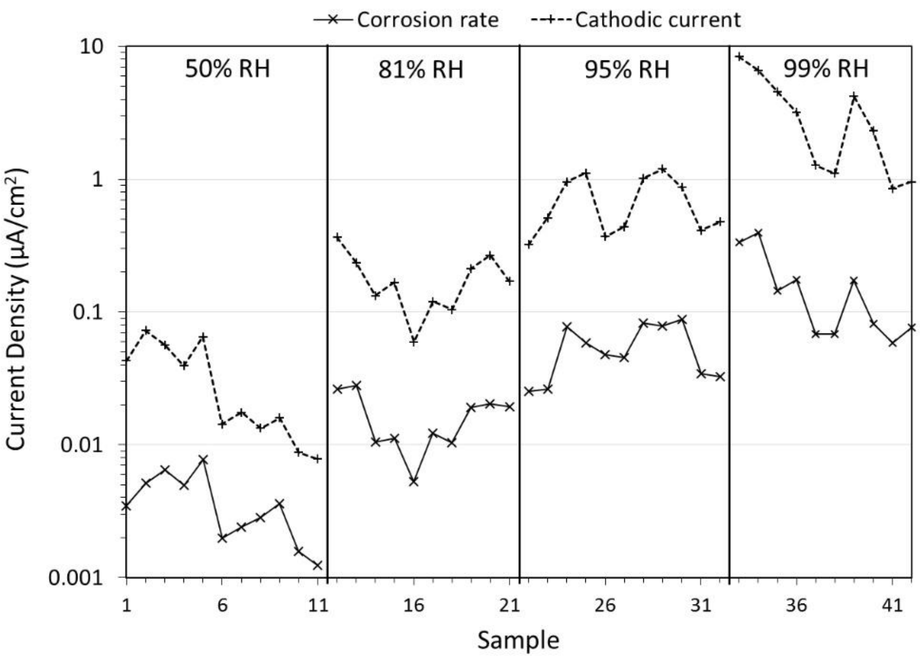

Supplementary Figure 4. Comparison between cathodic limiting current and corrosion rate, sample by sample. Corrosion rate and cathodic limiting current data, the x axis represents each single sample on which both cathodic current and corrosion rate were measured. By comparing the measured cathodic limiting current to the corrosion rate for the different samples it is possible to establish if the corrosion rate is limited by the cathodic reaction rate. It appears that the cathodic limiting current, regardless of the experimental variables, is always one order of magnitude higher than the corrosion rate, ruling out the possibility of cathodic control of the corrosion process. It also looks like the values of the two parameters vary with great consistence one with the other, sample by sample. This can be explained by the two processes having very much in common: both are electrochemical processes and both are strictly taking place at the electrode (embedded steel) surface.

Supplementary Note 1

Potential influence of oxygen concentration, iron concentration and pH on the corrosion rate

In electrochemical systems such as corrosion, the final reaction rate can be defined by the mixed potential theory [1,2,3]. The common representation of these systems is called Evans diagram (Supplementary Figure 5) and it shows the correlation between electrochemical potentials of the single semi-reactions and the logarithm of current density. In the present electrochemical process the final corrosion rate can be theoretically calculated from the specific parameters of each semi-reaction, which in this case are:

Cathodic semi-reaction: $O_2 + 2H_2O + 4e^- \rightarrow 4OH^-$ (1)

Anodic semi-reaction: $Fe \rightarrow Fe^{2+} + 2e^-$ (2)

Each of them is characterized by a reversible potential (E), an exchange current density (i^0) and a Tafel slope (b) that is the parameter correlating potentials and current for the same semi-reaction. They are expressed as [2,3]

$E = E^0 + \frac{RT}{nF} \log \frac{[Ox]}{[Rd]}$ Nernst Equation (3)

$i^0 = nFk[Ox]^{1-\alpha}[Rd]^{\alpha}$ (4)

$b = \pm \frac{RT}{\alpha nF}$ (5)

In the equations (3), (4) and (5) E^0 is the standard potential of the reaction, *[Ox]* and *[Rd]* stand for the concentration of oxidized and reduced specie of the same semi-reaction, *R* is the gas constant, *T* the temperature, *n* the number of exchanged electrons, *F* is the Faraday constant, *k* is the kinetic constant of the reaction and α is the charge transfer coefficient.

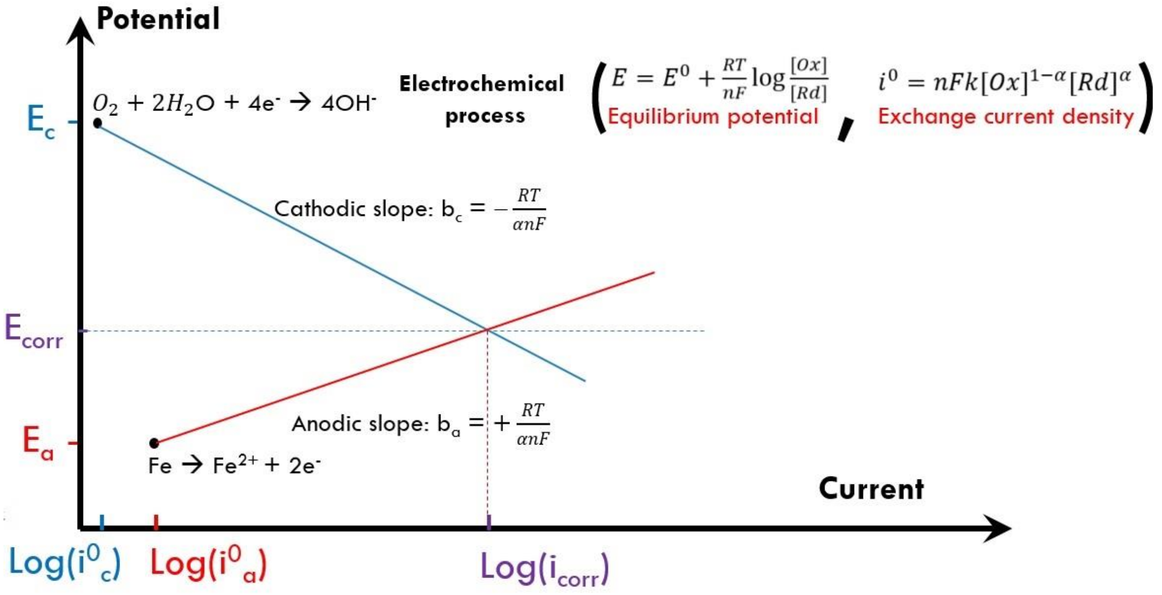

Supplementary Figure 5. Evans diagram. The corrosion electrochemical process is represented as normally intended in case of steel corrosion in carbonated concrete in a charge transfer controlled process. Cathodic and anodic processes are defined by their specific electrochemical parameters. The schematic representation is not in terms of absolute values; it represents an activation controlled electrochemical process is in terms of theoretical electrochemistry. The figure represents how an anodic and a cathodic process interact with each other in order to create a redox reaction, which is characterized by a mixed potential (E_{corr}) and a reaction rate (i_{corr}).

The equations describing cathodic and anodic straight lines (Supp. Fig. 5) can be written:

$$E_{corr} - E_{O_2/OH^-} = b_c(\log i^o{}_c - \log i_{corr}) \quad (6)$$

$$E_{corr} - E_{Fe/Fe^{2+}} = b_a(\log i^o{}_a - \log i_{corr}) \quad (7)$$

The theoretical corrosion rate can be, at this point, calculated as the intersection between cathodic and anodic functions, isolating first the intersection corrosion potential (E_{corr}) from both (6) and (7):

$$E_{corr} = E_{O_2/OH^-} + b_c(\log i^o{}_c - \log i_{corr}) = E_{Fe/Fe^{2+}} + b_a(\log i^o{}_a - \log i_{corr}) \quad (8)$$

$$E_{Fe/Fe^{2+}} - E_{O_2/OH^-} = b_c(\log i^o{}_c - \log i_{corr}) - b_a(\log i^o{}_a - \log i_{corr}) \quad (9)$$

From (9) the logarithm of the corrosion rate can be calculated based on the reactions parameters.

$$\log i_{corr} = \frac{E_{Fe/Fe^{2+}} - E_{O_2/OH^-} + b_c \log i^o{}_c - b_a \log i^o{}_a}{b_c - b_a} \quad (10)$$

Therefore the difference between the produced corrosion rates in two different conditions can be expressed as

$$\log i_{corr1} - \log i_{corr2} =$$

$$= \left(\frac{E_{O_2/OH^-} - E_{Fe/Fe^{2+}} + b_c \log i^o{}_c - b_a \log i^o{}_a}{b_c - b_a}\right)_1 - \left(\frac{E_{O_2/OH^-} - E_{Fe/Fe^{2+}} + b_c \log i^o{}_c - b_a \log i^o{}_a}{b_c - b_a}\right)_2 \quad (11)$$

Now it is possible to discuss the impact of species concentration in the pore solution on the corrosion rate and calculate what would be necessary to reach the experimentally measured corrosion rate span (factor 200). As it can be seen from equation (5), Tafel slopes do not depend on the species in solution so they will be considered constant. Equation (4) is a theoretical expression that cannot be used in practice because a single reaction can be composed of many steps [4,5,6], and typically the rate limiting step is unknown. Anyways this is a second order effect because the variation of experimentally measured exchange current densities was shown to impact on final corrosion rate of a maximum factor 0.3 [7].

Therefore the only parameter we can really evaluate, from a theoretical point of view, is the dependence of the reversible potentials on the species concentration in solution and their impact on the corrosion rate.

Starting with the cathodic reaction showed in eq. (1) the dependence of the cathodic reversible potential on the oxygen concentration is

$$E_{O_2/OH^-} = E^o{}_{O_2/OH^-} + \frac{RT}{nF} \ln \frac{[O_2]*[H_2O]^2}{[OH^-]^4} \quad (12)$$

From equation (11) all the parameters eliminate each other in the subtraction, but E_{O_2/OH^-} as dependent on two different oxygen concentration

$$\log i_{corr1} - \log i_{corr2} = \frac{E_{O_2/OH^-}{}_2 - E_{O_2/OH^-}{}_1}{b_c - b_a} \quad (13)$$

Substituting (12) in (13)

$$\log i_{corr1} - \log i_{corr2} = \frac{E^o{}_{O_2/OH^-} + \frac{RT}{nF} \ln \frac{[O_2]*[H_2O]^2}{[OH^-]^4}{}_2 - E^o{}_{O_2/OH^-} - \frac{RT}{nF} \ln \frac{[O_2]*[H_2O]^2}{[OH^-]^4}{}_1}{b_c - b_a} \quad (14)$$

The concentration of water is considered as 1

$$\log i_{corr1} - \log i_{corr2} = \frac{E^o{}_{O_2/OH^-} + \frac{RT}{nF}(\ln[O_2] - 4\ln[OH^-])_2 - E^o{}_{O_2/OH^-} - \frac{RT}{nF}(\ln[O_2] - 4\ln[OH^-])_1}{b_c - b_a} \quad (15)$$

The standard potentials get erased, $\frac{RT}{nF}$ can be calculated and the natural logarithm is transformed in base 10.

$$\log i_{corr1} - \log i_{corr2} = \frac{0.0145(\log[O_2] - 4\log[OH^-])_2 - 0.0145(\log[O_2] - 4\log[OH^-])_1}{b_c - b_a} \quad (16)$$

$$\log i_{corr1} - \log i_{corr2} = \frac{0.0145\log[O_2]_2 - 0.058pOH_2 - 0.0145\log[O_2]_1 + 0.058pOH_1}{b_c - b_a} \quad (17)$$

When the pH remains constant

$$\log i_{corr1} - \log i_{corr2} = \frac{0.0145\log[O_2]_2 - 0.0145\log[O_2]_1}{b_c - b_a} \quad (18)$$

As Tafel constants we use the most common values reported in [7], $b_a = 0.06$ and $b_c = -0.16$. Also using the maximum corrosion rate variation factor (200) it is obtained

$$\log 200 = 2.3 = \frac{0.0145(\log[O_2]_2 - \log[O_2]_1)}{-0.22} 34.9 = \log\frac{[O_2]_2}{[O_2]_1} \quad (19)$$

The oxygen concentration variation required to explain a corrosion rate difference in the order of a factor 200 can finally be calculated

$$\frac{[O_2]_2}{[O_2]_1} = 10^{34.9}$$

The same can be done when the influence of pH on the cathodic reaction is under examination, by taking back eq. (17) and solve it with respect to pH

$$2.3 = \frac{-0.058pOH_2 + 0.058pOH_1}{-0.22} \quad (20)$$

$$8.7 = pOH_1 - pOH_2 = 14 - pH_1 - 14 + pH_2 = pH_2 - pH_1 \quad (21)$$

The pH variation needed to have a 200 times different corrosion rate is

$$pH_2 - pH_1 = 8.7$$

Evaluating now the impact of iron concentration in the pore solution

$$\log i_{corr1} - \log i_{corr2} = \frac{E_{Fe/Fe^{2+}}{}_1 - E_{Fe/Fe^{2+}}{}_2}{b_c - b_a} \quad (22)$$

The expression of the anodic reaction reversible potential is

$$E_{Fe/Fe^{2+}} = E^o{}_{Fe/Fe^{2+}} + \frac{RT}{nF}\ln\frac{[Fe^{2+}]}{[Fe]} \quad (23)$$

Concentration of metallic iron is considered as 1, the calculations proceed as previously shown

$$\log i_{corr1} - \log i_{corr2} = \frac{E^o{}_{Fe/Fe^{2+}} + \frac{RT}{nF}\ln Fe^{2+}{}_1 - E^o{}_{Fe/Fe^{2+}} - \frac{RT}{nF}\ln Fe^{2+}{}_2}{b_c - b_a} \quad (24)$$

$$\log i_{corr1} - \log i_{corr2} = \frac{\frac{RT}{nF}\ln Fe^{2+}{}_1 - \frac{RT}{nF}\ln Fe^{2+}{}_2}{b_c - b_a} \quad (25)$$

$$2.3 = \frac{0.029\log Fe^{2+}{}_1 - 0.029\log Fe^{2+}{}_2}{-0.22}\, 2.3 = \frac{0.029(\log Fe^{2+}{}_1 - \log Fe^{2+}{}_2)}{-0.22}\, 17.5 = \log\frac{Fe^{2+}{}_1}{Fe^{2+}{}_2} \quad (26)$$

Finally the iron concentration difference that could explain the 200 factor in corrosion rate is

$$\frac{Fe^{2+}{}_1}{Fe^{2+}{}_2} = 10^{17.5}$$

The pore solution composition can explain the variation of corrosion rate from a relative humidity of 50% to 99% only by absurd variations of species concentration, such as:

- An oxygen concentration difference of 34.9 orders of magnitude;
- A pH difference of 8.7;
- An iron concentration difference of 17.5 orders of magnitude.

In conclusion the 200 factor of corrosion rate variation depending on the relative humidity, cannot be explained by an influence of the pore solution composition on the electrochemical process.

Supplementary references

[1] Wagner, C., & Traud, W. (2006). "On the Interpretation of Corrosion Processes through the Superposition of Electrochemical Partial Processes and on the Potential of Mixed Electrodes," with a Perspective by F. Mansfeld. *Corrosion*, *62*(10), 843-855.

[2] Hamann, C. H., Hamnett, A., & Vielstich, W. (2007). Electrochemistry, Second Edition.

[3] Bard, A. J., Faulkner, L. R., Leddy, J., & Zoski, C. G. (1980). *Electrochemical methods: fundamentals and applications* (Vol. 2). New York: wiley.

[4] Morgan, B., & Lahav, O. (2007). The effect of pH on the kinetics of spontaneous Fe (II) oxidation by O 2 in aqueous solution–basic principles and a simple heuristic description. *Chemosphere*, *68*(11), 2080-2084.

[5] Jovancicevic, V., & Bockris, J. M. (1986). The mechanism of oxygen reduction on iron in neutral solutions. *Journal of The Electrochemical Society*, *133*(9), 1797-1807.

[6] Katsumi Goto, Hiroki Tamura, Masaichi Nagayama (1969). The Mechanism of Oxygenation of Ferrous Ion in Neutral Solution. *Inorganic Chemistry*.

[7] Ge, J., & Isgor, O. B. (2007). Effects of Tafel slope, exchange current density and electrode potential on the corrosion of steel in concrete. *Materials and Corrosion*, *58*(8), 573-582.
